# Heritability of the morphology of optic nerve head and surrounding structures: The Healthy Twin Study

**DOI:** 10.1371/journal.pone.0187498

**Published:** 2017-11-16

**Authors:** Jong Chul Han, Hyeonyoung Ko, Seung Hoon Kim, Taekkwan Rhee, Seung Wan Nam, Sungsoon Hwang, Ga-In Lee, Joohon Sung, Yun-Mi Song, Changwon Kee

**Affiliations:** 1 Department of Ophthalmology, Samsung Medical Center, Sungkyunkwan University School of Medicine, Seoul, South Korea; 2 Department of Family Medicine, Samsung Medical Center, Sungkyunkwan University School of Medicine, Seoul, South Korea; 3 Department of Epidemiology, School of Public Health and Institute of Health and Environment, Seoul National University, Seoul, South Korea; ICAD Inc., UNITED STATES

## Abstract

**Background:**

Optic nerve head (ONH) and surrounding structures such as β-zone peripapillary atrophy (PPA) are important structures in glaucomatous pathogenesis. Thus, for understanding genetic components in glaucoma pathogenesis, it is necessary to evaluate the heritability of ONH and surrounding structures. The present study investigated the genetic influences on ONH and surrounding structures such as β-zone PPA and retinal vessels.

**Methods:**

A total of 1,205 adult twins and their family members (362 monozygotic (MZ) twin subjects (181 pairs), 64 dizygotic (DZ) twin subjects (32 pairs), and 779 singletons from 261 families), were part of the Korean Healthy Twin Study. ONH parameters including the vertical cup-to-disc ratio, the presence, the area and the location of β-zone PPA and the angular location of retinal vein were measured. The genetic influences on the structures were evaluated using variance-component methods.

**Results:**

The intraclass correlation coefficient (ICC) values of axial length were highest among the parameters. The ICCs of the area and location of PPA were similar to those of vertical cup-to-disc ratio. However, retinal vessel angular locations showed low ICC values even in MZ twins. After age and sex adjustment, for axial length, vertical cup-to-disc ratio, the presence, area and location of PPA, the estimated narrow-sense heritability was 0.85, 0.48, 0.76, 0.50 and 0.65 in the right eye and 0.84, 0.47, 0.72, 0.46 and 0.72 in the left eye, respectively. The estimated narrow-sense heritability of angular location of the superior and inferior vein was 0.17 and 0.12 in the right eye and 0.13 and 0.05 in the left eye, respectively.

**Conclusions:**

ONH and surrounding structures such as vertical cup-to-disc ratio and the presence, the area and the location of β-zone PPA seemed to be determined by the substantial genetic influence, whereas the venous angular location did not.

## Introduction

The optic nerve head (ONH) is considered as the important structure involved in glaucoma pathogenesis.[1] In order to diagnose glaucoma, the characteristics of the ONH, such as size, rim area and cup-to-disc ratio, need to be determined.[2] Changes in the structures surrounding the ONH, such as β-zone peripapillary atrophy (PPA), have been reported to be associated with glaucoma.[3–5] β-zone PPA is thought to be associated with retinal nerve fiber layer (RNFL) defect or visual field defect in glaucoma.[6,7] Recently, deep ONH structures such as lamina cribrosa and peripapillary sclera were reported to be important structures in glaucoma pathogenesis as a complete system.[8]

The sclera may be involved in the formation of the outer part of the eye and also be associated with the morphology of ONH and peripapillary atrophy.[9–11] In previous studies, the distributions of the structures move to the temporal direction with axial elongations.[9,12,13] Considering this may be related to the scleral structural changes with axial elongations, we speculated that ONH, PPA and the structures on the posterior pole such as retinal vessels may be affected by scleral changes related to axial length.

To date, researchers have sought to determine the inheritance of ocular structures. It is well known that axial length (AL) and spherical equivalent (SE) have strong inheritance.[14–17] Ocular inheritance was shown in anterior segment findings such as anterior chamber depth, astigmatism and corneal aberrations, and posterior segment characteristics such as disc size, cup size and cup-to-disc ratio.[14,17–20] However, few reports have shown the inheritance patterns of posterior structures like PPA morphologic characteristics and retinal vessel locations.

We intended to investigate whether genetic factors can contribute to the morphology of the ONH surrounding structures including PPA and retinal vessels in the posterior pole of the eye. Thus, we used a twin and family study to investigate whether morphologic characteristics of these structures can be inherited. In the present study, we investigated the parameters of vertical cup-to-disc ratio, and the presence, the area and the location of β-zone PPA. In addition, for understanding the heritability of the vessel distributions, the large retinal vein was used for measuring the angular location of the retinal vessels.

## Materials and methods

### The Healthy Twin Study

The study adhered to the tenets of the Declaration of Helsinki. Written informed consent was obtained from all participants. The study was approved by the institutional review board of the Samsung Medical Center [SMC2005-08-113].

The Healthy Twin Study is a nationwide prospective cohort study started in 2005 that has recruited Korean adult twins and their family members for genetic and epidemiologic study of complex diseases. Comprehensive ophthalmic examinations were conducted in the Department of Ophthalmology at the Samsung Medical Center in Seoul, South Korea, starting in 2007. The protocol and methodologic details have been published previously.[21] 1,346 participants were eligible for the present study. We excluded 141 participants due to a lack of family trees or fundus photo data. Finally, a total of 1,205 participants were included in the present study. There were 362 monozygotic (MZ) twin subjects (181 pairs), 64 dizygotic (DZ) twin subjects (32 pairs), and 779 singletons from 261 families. The singleton group was composed of the father, mother, and siblings of the twins, and MZ or DZ twins who participated in the study alone without their co-twin. Sixteen short tandem repeat marker kits (PerkinElmer’s AmpFlSTR, Norwalk, CT) were used to determine the zygosity of twins and to ascertain the family relationship of 67% of the twin pairs. For the remaining 33%, zygosity was determined based on a self-administered zygosity questionnaire developed by the research team, which showed a predictive value of more than 98% for MZ twins.[22]

### Optic nerve head, peripapillary atrophy and vein angular location measurement

Digital color fundus photographs (Nonmyd7; Kowa, Tokyo, Japan) were taken in all participants, and AL was measured by corneal touch A-scan ultrasonography (model 820; Allergan-Humphrey, San Leandro, CA, USA). Non-dilated refractions were measured in all participants with an autorefractor (Topcon AT; Topcon Corp., Tokyo, Japan). Data from both eyes of each subject were used and analyzed separately.

**Fig 1:**
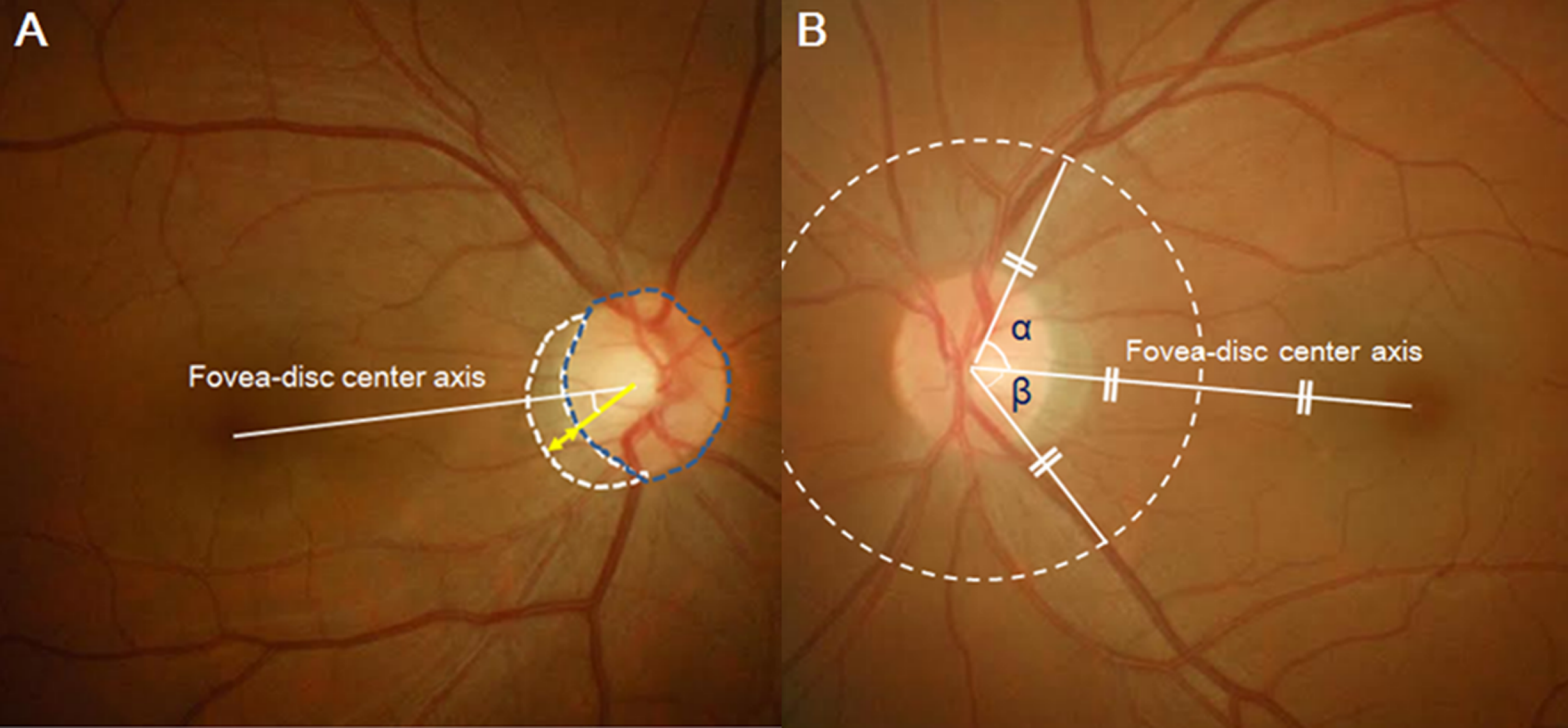
Parameter measurements in the present study. (A) Fovea-to-disc center distance connected the foveal center and optic disc center and was defined as the reference value (white line). The β-zone PPA was defined as the region of chorioretinal atrophy containing both visible sclera and choroidal vessels adjacent to the optic disc (dotted white line). Disc area was defined as the region in the inner margin of scleral ring around optic disc. PPA-to-disc area ratio was used as the modified PPA area in order to compensate for the magnification effect due to AL. The location of β-zone PPA was defined as the angle of the point of maximal radial extent from the reference line (yellow line). The point of maximum radial extent was the point on the temporal β-zone PPA margin where the radial extent of the β-zone PPA was maximal (yellow arrow). (B) The vein angular location was defined as the angle between the fovea-to-disc center (reference) and the largest temporal vein. To define the location of the vein, a circle with a radius half the distance of the fovea-to-disc center was drawn (dotted white line). The point at which the circle meets the vein was regarded as the location of vein (each end of the superior and inferior white lines). The angle between the location of superior vein and the reference was defined as the angular location of the superior vein (angle α). The angle between the location of the inferior vein and the reference was defined as the angular location of the inferior vein (angle β). The ratio of the superior vein angle and the inferior vein angle was defined as the vein angular location ratio.

Vertical cup-to-disc ratio and PPA area were measured by Image J software (version 1.52; National Institutes of Health, Bethesda, MD, USA). β-zone PPA was defined as the region of chorioretinal atrophy with both visible sclera and choroidal vessels adjacent to the optic disc.[23] Disc area was defined as the region in the inner margin of scleral ring around optic disc. The PPA-to-disc area ratio was used as the modified PPA area in order to compensate for the magnification effect according to AL.[6] The line connecting the center of fovea to the center of the optic disc was used as a reference line. The location of β-zone PPA was defined as the angle between the point of maximal radial extent and the reference line. The point of maximum radial extent was the point on the temporal β-zone PPA margin located the greatest distance from the disc center (Fig 1A).[24] The vein angular location was defined as the angle between the reference line and the largest temporal vein. To define the location of vein, a circle with a radius of half the distance of fovea-to-disc center length was drawn. The point at which the circle met the vein was regarded as the location of vein. The angle between the location of superior vein and the reference was defined as the angular location of superior vein. The angle between the location of inferior vein and the reference was defined as the angular location of inferior vein (Fig 1B). The ratio of the superior vein angle and the inferior vein angle was defined as the vein angular location ratio.

**Fig 2:**
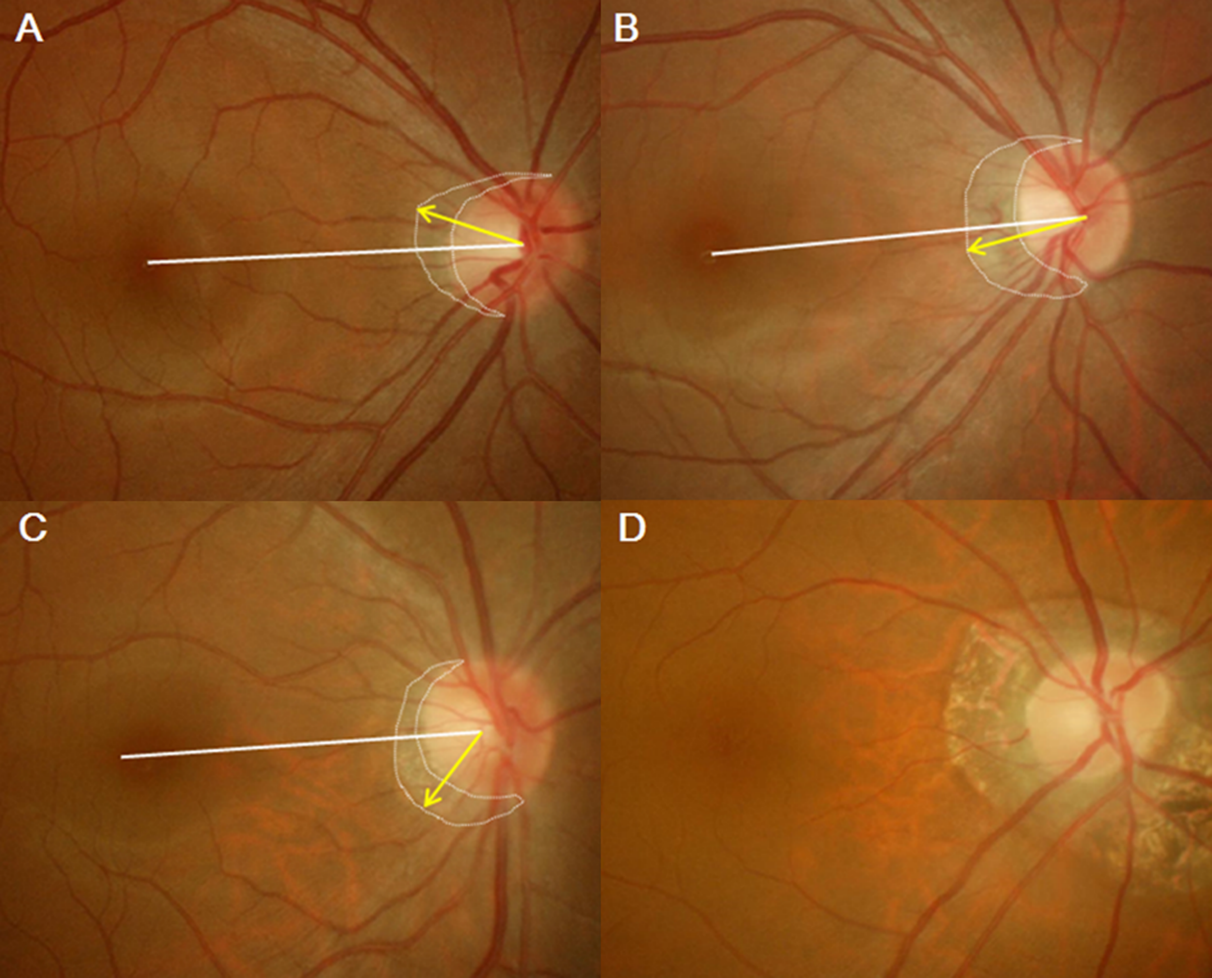
Classification of the location of β-zone PPA. (A) When the location of β-zone PPA was greater than 15 degrees superior, it was classified as superior. (B) When the location of β-zone PPA was less than 15 degrees, it was classified as temporal. (C) When the location of β-zone PPA was greater than 15 degrees inferior, it was classified as inferior. (D) When the β-zone PPA was located circumferentially, it was regarded as circumferential.

The locations of β-zone PPA were categorized superior, temporal, inferior and circumferential according to the PPA location. When the location of β-zone PPA was greater than 15 degrees superior, it was classified as superior. When the location of β-zone PPA is greater than 15 degrees inferior, it was classified as inferior. When the location of β-zone PPA was less than 15 degrees, it was classified as temporal.[25] When the β-zone PPA was located circumferentially, it was classified as circumferential (Fig 2). Two investigators (SHK and TKR) were involved to measure the parameters. The average values were used in the final analysis. In the presence and the location of PPA, the decision was made when two investigators had same opinions. If there were different opinions in the presence and the location of PPA, the final decisions were made through consensus agreement by the investigators including the third one (JCH).

### Statistical analysis

All analyses were carried out using each value of both eyes for each parameter. To determine the similarity of fundus parameters between each twin and their family members, we calculated ICC as the ratio of variance due to particular intra-familial relationship (i.e. between MZ twin) over total variance (the sum of the variance due to particular intra-familial relationship and the variance due to residual variance). We estimated the variance using the mixed linear regression model in which each family relationship was regarded as separate unit of random effects and, thus, the variance due to particular intra-familial relationship was the variance by random effect. If there is no correlation between the specific intra-familial relationship, then the variance by random effect is zero and ICC is zero. If there is complete correlation between the specific intra-familial relationship, then the variance by random effect can explain the whole variance and ICC is one. For this estimation, DZ twins were pooled with siblings because the genetic sharing within DZ twin pairs is similar to the genetic sharing between siblings and the number of DZ twin pair was too small to be separate.[21] Age and sex were adjusted for nontwins.

By using variance-component methods with the data of twins and their family members, we estimated heritability to quantify the genetic contributions to the ONH and surrounding structures.[26] A variance component approach was used to partition the total phenotypic variation (бp2) of each trait into its additive genetic component (бa2) and unmeasured environmental component (бe2). The key assumption of this model was that the effects of environmental factors are common to the members of a family and that the additive genetic (A), shared (C) and non-shared (E) environmental factors have independent and additive effects on the trait variance with the total residual variance being the sum of the additive and individual specific variance components (бp2 = бa2 + бc2 + бe2). We compared the model fitness of several models (ACE, AE, or E) using the likelihood ratio test (-2 log L1/L2, in which L1 and L2 are the maximized likelihoods of the restricted and the more general model, respectively). We chose the best fitting model by the chi-square approximation. We added chi-square statistic for the likelihood ratio test for heritability. By using the strength of twin and family design, we tried to estimate narrow-sense heritability that represents the proportion of the variance explained by additive genetic effects over the total phenotypic variance (бa^2^ / бp^2^), after adjusting for age, sex, intraocular pressure (IOP), and AL.

We evaluated ICC values between investigators to assess intraobserver and interobserver reliability of the continuous variables. In addition, the correlation between AL and PPA-to-disc area ratio and the correlation between AL and angular location of retinal vein were calculated using Pearson’s coefficient.

Descriptive statistics and ICC calculations were performed using statistical software (SAS, version 9.3; SAS Inc., Cary, NC). A variance-component model implemented in the Sequential Oligogenic Linkage Analysis Routines (SOLAR) software package (version 4.2.7; http://solar.txbiomedgenetics.org/; provided in the public domain by Texas Biomedical Research Institute) was used to calculate heritability.[27]

## Results

The characteristics of the study participants are shown in Table 1. A total of 1,205 right eyes and 1,198 left eyes of 1,205 participants were included in the analysis. The average age of the participants was 43.6 ± 13.63 years. There were 479 (39.7%) males and 726 (60.3%) females included in the study. The average IOPs were 14.73 ± 2.98 mmHg for the right eye and 14.80 ± 3.08 mmHg for the left eye. The average SEs were -0.55 ± 2.36 diopters and -0.53 ± 2.35 diopters for the right and left eyes, respectively. The average ALs were 23.98 ± 1.40 mm and 23.96 ± 1.40 mm for the right and left eyes, respectively. The average vertical cup-to-disc ratios were 0.37 ± 0.10 and 0.37 ± 0.10 for the right and left eyes, respectively. The PPA-to-disc area ratio was 0.17 ± 0.34 on the right and 0.15 ± 0.32 on the left. Among the participants, 562 (46.6%) right eyes and 526 (43.9%) left eyes showed β-zone PPA. The β-zone PPAs of right eyes located superiorly in 49 eyes (8.7%), temporally in 376 eyes (66.9%), inferiorly in 112 eyes (19.9%), and circumferentially in 25 eyes (4.4%). The β-zone PPAs of left eyes located superiorly in 56 eyes (10.6%), temporally in 320 eyes (60.8%), inferiorly in 131 eyes (24.9%), and circumferentially in 19 eyes (3.6%). The average angular locations of a superiorly located vein were 71.56 ± 13.15 degrees and 73.89 ± 13.87 degrees in the right and the left eye, respectively. The average angular locations of inferiorly located vein were 65.60 ± 15.13 degrees and 64.66 ± 14.90 degrees in the right and the left eye, respectively. The average angle ratio was 1.15 ± 0.33 and 1.20 ± 0.34 in right and left eye, respectively. Intraobserver and interobserver ICCs were high (0.84 to 0.94) across all parameters (Table 2).

**Table 1:** Characteristics of study subjects and their eyes.

	Right eye (n = 1205)	Left eye (n = 1198)
**Age (years)**	43.63 ± 13.63	
**Sex**		
**Men, n (%)**	479 (39.7)	
**Women, n (%)**	726 (60.3)	
**Monozygotic twins, n (%)**	362 (30.0)	
**Dizygotic twins, n (%)**	64 (5.3)	
**Singletons, n (%)**	779 (64.6)	
**Intraocular pressure (mmHg)**	14.73 ± 2.98	14.80 ± 3.08
**Spherical equivalent (diopter)**	-0.55 ± 2.36	-0.53 ± 2.35
**Axial length (mm)**	23.98 ± 1.40	23.96 ±1.40
**Vertical cup-to-disc ratio**	0.37 ± 0.10	0.37 ± 0.10
**Presence of PPA, n (%)**	562 (46.6)	526 (43.9)
**PPA to disc area ratio**	0.17 ± 0.34	0.15 ± 0.32
**Location of PPA**		
**Superior, n (%)**	49 (8.7)	56 (10.6)
**Temporal, n (%)**	376 (66.9)	320 (60.8)
**Inferior, n (%)**	112 (19.9)	131 (24.9)
**Circumferential, n (%)**	25 (4.4)	19 (3.6)
**Angular location (superior vein) (°)**	71.56 ± 13.15	73.89 ± 13.87
**Angular location (inferior vein) (°)**	65.60 ± 15.13	64.66 ± 14.90
**Angle ratio (superior/inferior)**	1.15 ± 0.33	1.20 ± 0.34

PPA = peripapillary atrophy.

Values are shown in mean ± standard deviation unless otherwise indicated.

**Table 2:** Intraobserver and interobserver reliability in fundus parameters.

Variables	Intraobserver ICC (95% CI)	Interobserver ICC (95% CI)
Vertical cup-to-disc ratio	0.86 (0.72–0.94)	0.84 (0.69–0.92)
PPA-to-disc area ratio	0.94 (0.86–0.98)	0.93 (0.85–0.97)
Vessel angle of superior vein	0.90 (0.78–0.96)	0.91 (0.81–0.95)
Vessel angle of inferior vein	0.91 (0.79–0.95)	0.88 (0.76–0.94)
Angle ratio (superior/inferior)	0.90 (0.82–0.95)	0.93 (0.86–0.97)

ICC = intraclass coefficient; PPA = peripapillary atrophy.

Table 3 shows the ICC of AL and fundus parameters within each pair of intra-familial relationship after adjusting for covariates. In the eye of MZ twin pairs, the ICC of AL was highest (0.87 in the right eye and 0.88 in the left eye), followed by vertical cup-to-disc ratio (0.67 in the right eye and 0.65 in the left eye), PPA-to-disc area ratio (0.53 in the right eye and 0.49 in the left eye), PPA location (0.58 in the right eye and 0.60 in the left eye), angular location of the superior retinal vein (0.25 in the right eye and 0.23 in the left eye), angular location of the inferior retinal vein (0.18 in the right eye and 0.14 in the left eye), and angle ratio (0.13 in the right eye and 0.13 in the left eye). The ICCs within MZ twin pairs were around two times higher than the ICCs from father-offspring pairs, mother-offspring pairs, or sibling pairs for AL and most of the fundus parameters. There was no substantial difference in the ICC estimates between right and left eye.

**Table 3:** Correlations of fundus parameters between family members.

Measurement	Intrafamilial relationship			
	MZ twin pairs	Father-offspring pairs	Mother-offspring pairs	Pooled DZ and sibling pair
Right				
Axial length*	0.87 (0.83–0.90)	0.32 (0.22–0.41)	0.37 (0.29–0.44)	0.41 (0.32–0.49)
Vertical cup-to-disc ratio^‡^	0.67 (0.58–0.74)	0.38 (0.29–0.47)	0.40 (0.33–0.47)	0.37 (0.28–0.46)
PPA to disc area ratio^‡^	0.53 (0.42–0.63)	0.23 (0.13–0.32)	0.14 (0.06–0.22)	0.26 (0.16–0.35)
PPA location^†^	0.58 (0.48–0.69)	0.12 (0.04–0.20)	0.11 (0.05–0.17)	0.19 (0.12–0.27)
Angular location of superior vein^‡^	0.25 (0.10–0.38)	0.08 (-0.03–0.18)	0.11 (0.03–0.20)	0.13 (0.02–0.22)
Angular location of inferior vein^‡^	0.18 (0.03–0.32)	0.09 (-0.01–0.19)	0.06 (-0.03–0.14)	0.07 (-0.03–0.17)
Angle ratio (superior/inferior)^‡^	0.13 (-0.01–0.27)	-0.02 (-0.12–0.09)	0.03 (-0.06–0.11)	-0.02 (-0.12–0.09)
Left				
Axial length*	0.88 (0.84–0.91)	0.33 (0.23–0.41)	0.39 (0.31–0.46)	0.46 (0.37–0.53)
Vertical cup-to-disc ratio^‡^	0.65 (0.54–0.74)	0.37 (0.27–0.46)	0.35 (0.27–0.42)	0.31 (0.22–0.41)
PPA to disc area ratio^‡^	0.49 (0.37–0.59)	0.10 (0.00–0.20)	0.14 (0.05–0.22)	0.20 (0.10–0.29)
PPA location^†^	0.60 (0.50–0.70)	0.03 (-0.05–0.10)	0.13 (0.07–0.19)	0.11 (0.03–0.18)
Vertical cup-to-disc ratio^‡^	0.55 (0.44–0.64)	0.27 (0.17–0.36)	0.35 (0.27–0.42)	0.31 (0.22–0.41)
Angular location of superior vein^‡^	0.23 (0.09–0.37)	0.05 (-0.05–0.15)	0.12 (0.03–0.20)	0.15 (0.05–0.24)
Angular location of inferior vein^‡^	0.14 (0.00–0.28)	0.09 (-0.01–0.19)	0.02 (-0.07–0.10)	-0.01 (-0.11–0.09)
Angle ratio (superior/inferior)^‡^	0.13 (-0.02–0.27)	0.04 (-0.06–0.14)	0.01 (-0.08–0.09)	0.00 (-0.10–0.10)

Abbreviations: DZ, dizygotic twins; MZ, monozygotic twins; PPA, peripapillary atrophy.

* Estimated by the intra-class correlation coefficient (95% CI) after adjusting for age and sex.

† Estimated by the Cohen’s kappa coefficient (95% CI).

‡ Estimated by the intra-class correlation coefficient (95% CI) after adjusting for age, sex, intraocular pressure, and axial length.

Table 4 shows the heritability of AL and fundus parameters and fitted variance component model. The best fitting model was an additive genetic and unique environment (AE) effects model for AL, presence of PPA, PPA-to-disc area ratio and PPA location in both eyes and an additive genetic, shared environment, and unique environment (ACE) effects model for vertical cup-to-disc ratio in both eyes. The best fitting model was an ACE effects model for angular location of superior vein (right eye) and an AE effects model for angular location of inferior vein (right eye) and superior vein (left eye). The unique environment (E) effects model was the best fitting model for angular location of inferior vein (left eye) and the angle ratio in both eyes. Age and sex-adjusted heritability of the right (left) eye was 0.85 (0.84) for AL, 0.48 (0.47) for vertical cup-to-disc ratio, 0.76 (0.72) for presence of PPA, 0.50 (0.46) for PPA-to-disc area ratio, 0.65 (0.72) for PPA location, 0.17 (0.13) for superior vein angular location, 0.12 (0.05) for inferior vein angular location, and 0.05 (0.06) for angle ratio. Age, sex, IOP, and axial length adjusted heritability of the right (left) eye was 0.48 (0.47) for vertical cup-to-disc ratio, 0.81 (0.77) for presence of PPA, 0.51 (0.45) for PPA-to-disc area ratio, 0.66 (0.71) for PPA location, 0.14 (0.14) for superior vein angular location, 0.12 (0.02) for inferior vein angular location, and 0.05 (0.06) for angle ratio.

**Table 4:** Heritability (*h*^*2*^) of the fundus parameter measurements.

	Variance component (Age, sex, IOP and AL adjusted)			Best-fitting Model	Chi-square statistic	*h*^*2*^± SE	
	A	C	E			Age & sex adjusted	Multivariable adjusted*
Right							
Axial length	0.85^*^	0	0.15	AE	358.20	0.85 ± 0.02	
Vertical cup-to-disc ratio	0.48^*^	0.13^‡^	0.39	ACE	43.31	0.48 ± 0.08	0.48 ± 0.08
Presence of PPA	0.81^*^	0	0.19	AE	113.27	0.76 ± 0.07	0.81 ± 0.06
PPA to disc area ratio	0.51^*^	0	0.49	AE	103.78	0.50 ± 0.05	0.51 ± 0.05
PPA location	0.66^*^	0	0.34	AE	28.28	0.65 ± 0.10	0.66 ± 0.10
Angular location of superior vein	0.14^†^	0.02^‡^	0.84	ACE	3.69	0.17 ± 0.07	0.14 ± 0.07
Angular location of inferior vein	0.12^†^	0	0.88	AE	8.07	0.12 ± 0.07	0.12 ± 0.05
Angle ratio (superior/inferior)	0.05	0	0.95	E	1.34	0.05 ± 0.05	0.05 ± 0.05
Left							
Axial length	0.84^*^	0	0.16	AE	361.60	0.84 ± 0.02	
Vertical cup-to-disc ratio	0.37^*^	0.17^*^	0.36	ACE	25.85	0.37 ± 0.08	0.37 ± 0.08
Presence of PPA	0.77^*^	0	0.23	AE	90.20	0.72 ± 0.07	0.77 ± 0.07
PPA to disc area ratio	0.45^*^	0	0.55	AE	83.97	0.46 ± 0.05	0.45 ± 0.05
PPA location	0.72^*^	0	0.28	AE	47.21	0.72 ± 0.06	0.71 ± 0.07
Angular location of superior vein	0.14^*^	0	0.86	AE	11.93	0.13 ± 0.05	0.14 ± 0.05
Angular location of inferior vein	0.02	0.06	0.92	E	0.10	0.05 ± 0.08	0.02 ± 0.08
Angle ratio (superior/inferior)	0.06	0.003	0.937	E	0.54	0.06 ± 0.08	0.06 ± 0.08

A = additive genetic effects; AL = axial length; C = shared environment effects; E = unique environment effects; IOP = intraocular pressure; PPA = peripapillary atrophy.

* *P* < 0.001

^†^ *P* < 0.01

^‡^ *P* < 0.05.

**Fig 3:**
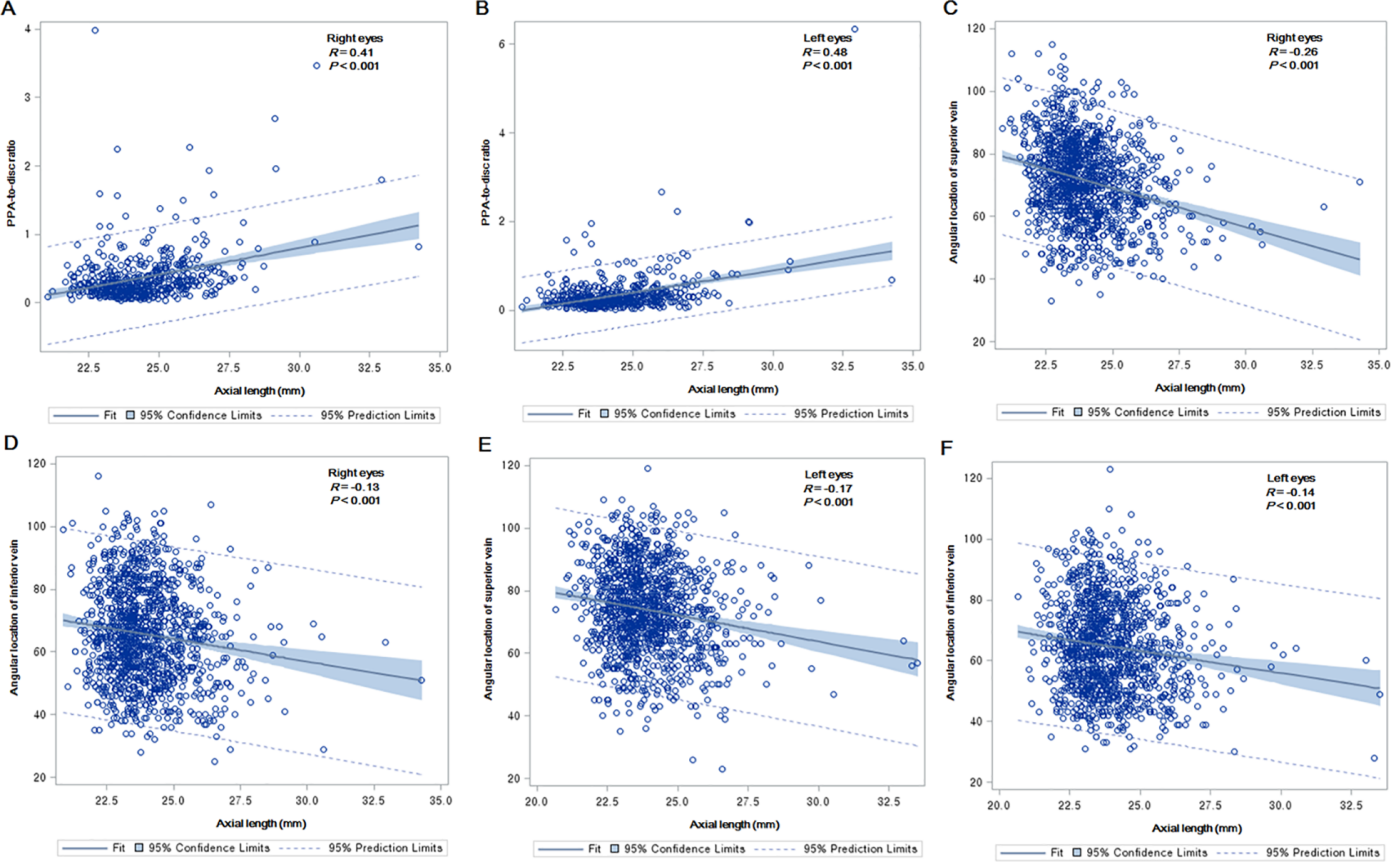
Correlations between axial length (AL) and peripapillary atrophy (PPA)-to-disc ratio and angular location of retinal vein. (A), (B) Positively linear correlations were shown between AL and PPA-to-disc ratio. (C-F) Negatively linear correlations were shown between AL and angular locations of retinal vessels, but the correlations seemed weak.

PPA-to-disc ratio was positively correlated with AL (Right eye, *R* = 0.41, *P* < .001; left eye, *R* = 0.48, *P* < .001) Vessel angles of superior vein and inferior veins were weak negatively correlated with AL (Right eye, *R* = -0.26 at superior vein, *P* < .001; *R* = -0.13 at inferior vein, *P* < .001; left eye, *R* = -0.17 at superior vein, *P* < .001; *R* = -0.14 at inferior vein, *P* < .001) (Fig 3).

## Discussion

Heritability is defined as the degree to which individual genetic variation accounts for phenotypic variation seen in a population. The twin study can offer an opportunity for dividing the genetic and environmental component in the phenotypic variance.[28] The present study demonstrated that AL, vertical cup-to-disc ratio and the presence of PPA were highly heritable in Koreans. This result is consistent with previous studies.[14,20,29] Furthermore, we confirmed that the area and location of PPA are significantly and highly heritable. However, the angular location of retinal vein was found to be under weak or insignificant genetic effect.

Myopia is strongly influenced by genetic factors.[14,15,30] In the present study, the heritability of AL was highest among the parameters. In addition, significant inheritance was shown in vertical cup-to-disc ratio. Previous studies showed substantially high heritability of ONH structures like optic disc size, cup size and cup-to-disc ratio. Hewitt et al showed higher heritability for disc area and cup size than for ONH vascular structures.[31] Drobnjak et al showed the high heritability of optic disc and cup size through a sample of 55 MZ and 50 DZ healthy twin pairs.[20] He et al studied 355 MZ and 202 DZ twin pairs in the Guangzhou Twin Registry to show that ONH parameters like disc area, cup area and cup-to-disc ratio have significant inheritance.[28]

**Fig 4:**
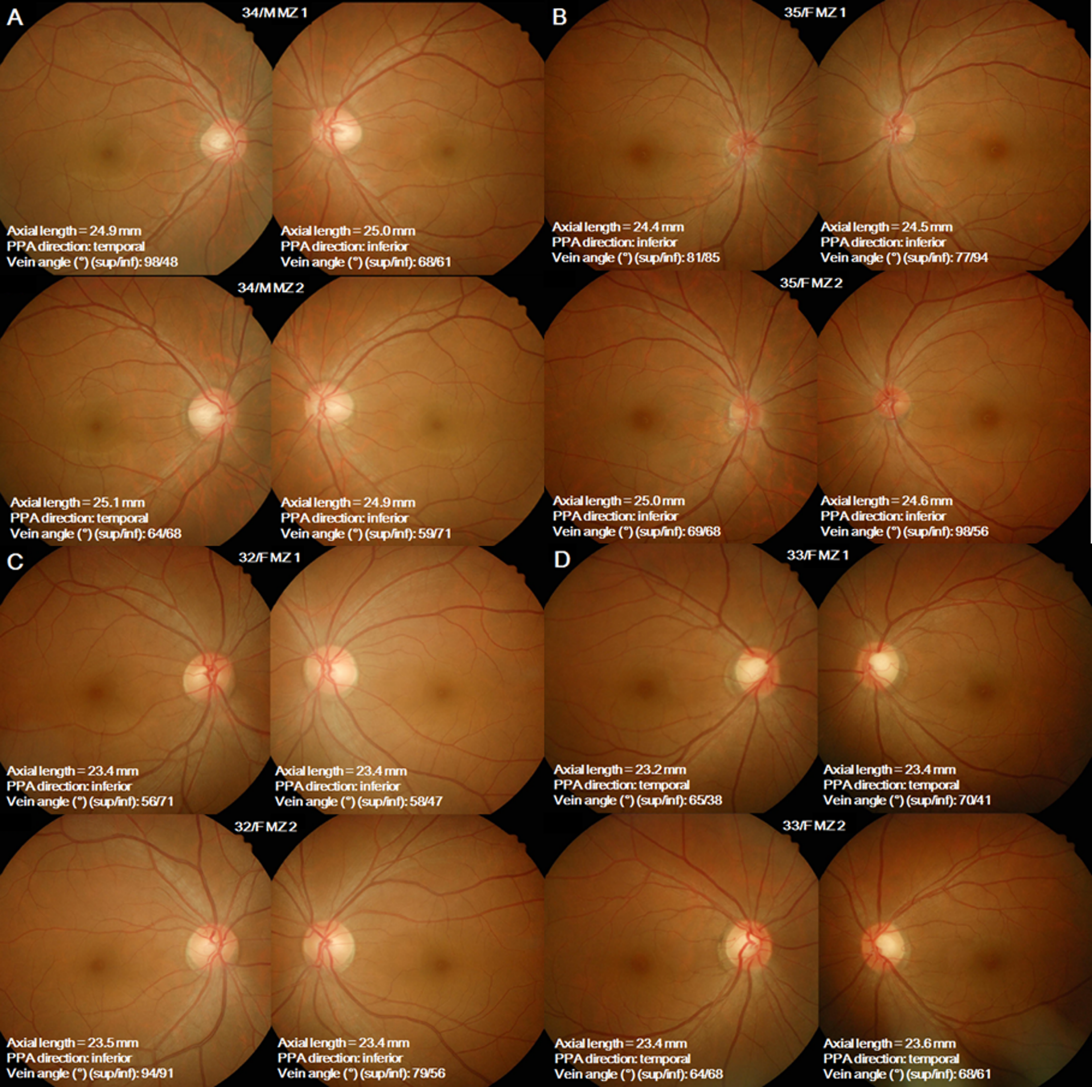
Representative cases. (A, B) Monozygote (MZ) twins with axial length (AL) > 24 mm. (C, D) MZ twins with AL < 24 mm. The areas and the locations of PPA are similar between MZ twins. Though the PPA area was shown to be correlated with AL, there were some exceptions that are thought to be associated with familial inheritance. MZ twin eyes with shorter AL (D) show greater PPA areas compared to the other MZ twin eyes with longer AL (A). The angular locations of retinal veins show various values even between MZ twins.

Healey et al not only demonstrated that genetic effects are important in determining ONH parameters like optic disc, cup, and rim areas,[19] but also showed that PPA could be inheritable.[29] PPA may be associated with ONH deformation with myopic elongation, and, as such, it is plausible that PPA is larger with a longer AL.[9,32] However, they did not demonstrate the inheritance of the area and the locations of PPA. The present study showed the significantly high heritability of the area and the location of PPA. Furthermore, the presence, the area and the location of PPA were also significantly heritable, even after adjusting for AL (Fig 4). This suggests that PPA in myopia is not just the result of axial elongation, but that PPA can be affected by familial inheritance. Though each eyeball grows similarly, ONH deformation may differ between eyes according to familial inheritance. In the previous studies, PPA was shown to be larger and more frequently found in glaucoma eyes[3,33] and the PPA location was reported to be spatially associated with the location of visual field defect and RNFL defects.[6,34,35] The heritability of the presence, the area and the location of PPA may offer insight into how glaucoma is inherited, and also may partly explain why some myopic eyes undergo glaucomatous damage, while others do not.

Previous studies have also investigated the heritability of the retinal vessel characteristics. Huntzinger et al demonstrated that vascular tortuosity seems to have higher heritability than other features such as vessel length, branching points, and number of vessels crossing the optic disc margin.[36] Taarnhøj et al showed that there are inheritance patterns in the presence of cilioretinal arteries[37] and retinal vessel diameter.[38] However, there is a lack of information on the heritability of the locations of retinal vessels.

In the present study, the angular location of the retinal vein seemed to have weak or absent inheritance. It remains unclear why the ONH and PPA have high heritability, whereas the angular location of retinal vein does not. We hypothesized two possible reasons on this. First, the amount of change around ONH is smaller than change surrounding posterior pole structures, like retinal vessels. Because continuous growth and remodeling of the sclera can occur with axial growth near the posterior pole of the eye, that area may have a higher likelihood of change.[39] We propose that if more change occurs, there is a higher chance of variation. Second, we speculated that normal formation and location of retinal vessels can be affected not only by AL, but also by physiologic conditions, such as the concentration of oxygen at the neonatal period.[40] Though twins share common genetic component, retinal vasculature can differ between them based on their physiologic environment at an early age.

There were significant correlations between PPA area and AL. This may be because PPA and AL are affected by the scleral structural changes.[41] We speculated that, if the vessel distributions are correlated with AL, the angular location of retinal vein may be used as the surrogate to reflect the scleral changes on the posterior pole. Previous studies using optical coherence tomography (OCT) showed that the RNFL distribution moves temporally with myopic elongation.[12,13] However, the correlations were weak even though the angular locations of retinal veins were inversely correlated to axial elongation. Thus, the angular location of retinal vein hardly seems to be used as the surrogate to reflect scleral changes of the posterior pole.

There are several limitations in the present study. First, the present study cannot guarantee whether included twins satisfied “equal environment” assumption. Previously, it was known that MZ twins may share more similar post-natal environment compared to DZ twins.[42] Furthermore, the study did not offer environmental information in detail. Thus, the study could not offer the reason for the inter-twins difference in the heritability of optic disc and surrounding structures. Second, given that the subjects were included from a Korean population and ONH characteristics could be different between ethnic groups, the findings from our study may not be generalized to other population. Third, the proportion of the PPA was greater than the previous report.[29] This may be because the present twin study included greater number of myopic eyes than previous reports. Fourth, the present study used only fundus photos to measure the characteristics of ONH and surrounding structures. In previous study, it was known that the ONH surface was not consistent with the deep Bruch’s membrane opening.[43] In further studies, the heritabilities of the deep ONH structures using OCT are warranted. Fifth, the angular locations of retinal vein was measured the angle of retinal vein just at one location. The present study results associated with the retinal vein should be interpreted with attention.

In conclusion, ONH characteristics such as vertical cup-to-disc ratio and the presence, the area and the location of PPA seemed to have substantial genetic inheritance patterns, whereas the venous angular location did not. These results showed phenotype inheritance of the ONH and surrounding structures, which may lead to further studies for genes involved in the characteristics of the ONH and surrounding structures.
